# Simple steps to develop trial follow-up procedures

**DOI:** 10.1186/s13063-016-1155-1

**Published:** 2016-01-15

**Authors:** Ona McCarthy, Rebecca S. French, Ian Roberts, Caroline Free

**Affiliations:** Department of Population Health, London School of Hygiene and Tropical Medicine, Keppel St, London, WC1E 7HT United Kingdom; Department of Social and Environmental Health Research, London School of Hygiene and Tropical Medicine, 15-17 Tavistock Place, London, WC1H 9SH UK

**Keywords:** Young people, Response rates, Follow-up, Postal questionnaire, Postal test kit, Randomised controlled trial, Sexually transmitted infections, Sexual health, mHealth

## Abstract

**Background:**

Loss to follow-up in randomised controlled trials reduces statistical power and increases the potential for bias. Almost half of all trials fail to achieve their follow-up target. Statistical methods have been described for handling losses to follow-up and systematic reviews have identified interventions that increase follow-up. However, there is little guidance on how to develop practical follow-up procedures. This paper describes the development of follow-up procedures in a pilot randomised controlled trial of a sexual health intervention that required participants to provide and return questionnaires and chlamydia test samples in the post. We identified effective methods to increase follow-up from systematic reviews. We developed and tested prototype procedures to identify barriers to follow-up completion. We asked trial participants about their views on our follow-up procedures and revised the methods accordingly.

**Results:**

We identified 17 strategies to increase follow-up and employed all but five. We found that some postal test kits do not fit through letterboxes and that that the test instructions were complicated. After identifying the appropriate sized test kit and simplifying the instructions, we obtained user opinions. Users wanted kits to be sent in coloured envelopes (so that they could identify them easily), with simple instructions and questionnaires and wanted to be notified before we sent the kits. We achieved 92 % (183/200) overall follow-up for the postal questionnaire at 1 month and 82 % (163/200) at 12 months. We achieved 86 % (171/200) overall follow-up for the postal chlamydia test at 3 months and 80 % (160/200) at 12 months.

**Conclusions:**

By using established methods to increase follow-up, testing prototype procedures and seeking user opinions, we achieved higher follow-up than previous sexual health trials. However, it is not possible to determine if the increase in response was due to our follow-up procedures.

**Trial registration:**

Current Controlled Trials ISRCTN02304709 Date of registration: 27 March 2013.

**Electronic supplementary material:**

The online version of this article (doi:10.1186/s13063-016-1155-1) contains supplementary material, which is available to authorized users.

## Background

Loss to follow-up in randomised controlled trials (RCTs) reduces statistical power and increases the potential for bias. Bias can occur when loss to follow-up is associated with the outcomes, such as when those lost have poorer outcomes then those retained. In order to explore the potential bias introduced, a range of plausible assumptions about outcomes in those lost to follow-up can be made [1]. A systematic review of trials reported in top medical journals found that when different plausible assumptions are made, the interpretation of trial results could change [1].

Almost half of all trials fail to achieve their follow-up target [2] and achieving high follow-up when collecting data on sensitive topics such as sexual health is particularly challenging. In sexual health research, response rates for self-reported data and test kits have been relatively low in both RCTs and surveys. The National Survey of Sexual Attitudes and Lifestyles (Natsal) study in the UK, a probability sample household survey, achieved 57.7 % response rate for face-to-face interviews and 60 % response for urine samples requested (not all responders were asked to provide a sample) [3]. A UK cross-sectional population-based study reported an uptake of chlamydia postal screening of 31.5 % in people aged 16–24 (the chlamydia screening studies ‘ClaSS’ project) [4, 5]. A pilot trial of a sexual health website intervention for young people (‘Sexunzipped’) achieved 45 % follow-up using chlamydia postal test kits and 72 % for self-reported data at 3 months [6].

Effective interventions for increasing follow-up have been developed and evaluated [7–12]. However, there is little guidance on how to develop practical follow-up procedures. Specifically, there is no guidance for developing follow-up procedures for RCTs on sensitive topics.

### Intervention development and pilot trial

The National Institute for Health Research Health Technology Assessment Programme (NIHR HTA) commissioned us to develop a mobile phone-based intervention to promote safer sex behaviour in young people aged 16–24 in the UK and to conduct a pilot RCT of the intervention. Key parameters for judging the success of the research were the acceptability of the intervention and the feasibility of recruitment and follow-up in a RCT. We conducted formative research, which included focus group discussions (FGD) to inform the intervention content.

In the pilot trial we recruited participants from seven sexual health services in London, Manchester, Cambridgeshire, Norfolk, Maidstone and Hull. People aged 16–24 who had had a recent positive chlamydia test result or had reported unsafe sex in the last year (defined as more than one partner and at least one occasion of unprotected sex) were eligible to take part. Recruitment staff invited eligible participants to take part in the study either at the site or by telephone. Participants provided informed consent either written or through the secure online database. Participants were randomised by a remote randomisation service, ensuring allocation concealment. Intervention or control group messages were sent from our automated system according to allocation. The intervention consisted of up to 63 text messages and employed 12 behaviour change techniques [13]. The messages were tailored according to infection status at enrolment and gender. Control participants received monthly messages about the importance of trial participation. We collected self-reported sexual health outcome data using postal questionnaires at 1 month and 12 months and chlamydia postal testing kits at 3 months and 12 months. Participants had the option of completing follow-up by attending the clinic or completing the questionnaire online, by text message or email.

We randomised 200 people between 9 September 2013 and 26 November 2013. Sixty-six percent of eligible participants joined the trial. The intervention development and pilot trial details are reported elsewhere [14].

In order to minimise bias in our trial we aimed to maximise follow-up. In this paper we describe the approach we used to develop the pilot trial follow-up procedures.

## Methods

Ethical approval for the intervention development phase of the project was granted by NRES Committee London-Bentham on 6 November 2012 (REC reference number 12/LO/1329). Ethical approval for the pilot trial was granted by NRES Committee South East Coast-Surrey on 26 July 2013 (REC reference number 13/LO/1001).

We developed our trial follow-up procedures in three steps.

### Step 1. Identifying evidence-based effective strategies to increase follow-up in trials

We searched the Cochrane Library for systematic reviews of trials of interventions designed to increase follow-up in research. We identified methods for which there was there was evidence of success in increasing response to postal follow-up requests in trials. We developed prototype follow-up procedures incorporating the effective strategies identified.

### Step 2. Testing prototype follow-up procedures and materials

We obtained sample postal test kits routinely used in our trial recruitment sites (Cambridgeshire, Manchester and London) and test kits used in the NHS chlamydia postal testing services. We measured London letterboxes on central London streets and measured the test kits to identify those that would fit through the smallest letter boxes.

We attempted to follow the instructions that were included in the postal test kit that would fit through the smallest letter box. Based on this experience, we generated ideas on how to make the follow-up process easier. We generated prototype test kits including combinations of the original and simplified materials. We gave test kits to volunteers from the Clinical Trials Unit (CTU) and asked them to provide feedback on the original and simplified materials. We also consulted with experts in sexual health regarding the questionnaire design and follow-up procedures.

### Step 3. Consulting with users

We asked young people aged 16–-24 for their views regarding the questionnaires and follow-up procedures in four of the eight FGD convened to inform the development of the intervention. We recruited FGD participants from community sexual and reproductive health services in South-east London, Greater Manchester and rural Cambridgeshire. Clinic staff used convenience and snowball sampling methods to recruit participants. The facilitators (OM, CF, and RF) provided verbal and written study information and obtained informed written consent from participants. All discussions were audio-recorded. We explored the participants’ preferences regarding intervention content and trial follow-up procedures.

As part of the pilot trial, we conducted phone interviews with participants [15]. The pilot trial consent form included an optional tick box where the participant could consent to be contacted regarding participation in the interview study. One hundred and sixty-seven participants consented to be contacted for an interview. We purposively sampled pilot trial participants 2 to 3 weeks after enrolment so that the interview sample varied according to age, gender, sexually-transmitted infection (STI) test result at enrolment, location (urban/rural) and whether they had been allocated to the intervention or control group. We gave participants verbal and written information about the study and obtained informed written consent either by email or text message. All the participants that we contacted agreed to participate (*n* = 20). We conducted follow-up phone interviews with the same participants after sending the 3-month test kits. We asked participants for their views on the trial materials and follow-up procedures. All interviews were audio-recorded. We conducted a descriptive analysis after taking notes on the recordings.

## Results

### Step 1. Effective strategies to increase postal follow-up

The follow-up strategies where there is evidence of increasing the odds of response are reported in Additional file 1 [7–12, 16]. We employed all but five of the 17 strategies.

### Step 2. Testing the prototype follow-up procedures

Only one test kit would fit through the smallest London letterbox (approximately 19 cm × 2.5 cm). This kit was provided by a laboratory diagnostic company, which contained pre-packaged components. Contents of the test kit included nine items (see Additional file 2). The components in the test kit included non-essential items, which we removed. The instructions for women could be confusing because the kit contained both the swab and the urine tube but they were required to provide only one sample.

Of the 12 CTU volunteers who were asked to provide a urine sample in the pouch, only one was able to use it successfully. Female volunteers did not express a preference for providing urine or vaginal swab samples. They preferred the simplified instructions and suggested that we include a statement in the postal letters about the importance of their participation so that they would feel ‘proud’ about doing something good.

Experts in sexual health questionnaire design suggested that we order the key questions, those on treatment and sexual health behaviour, first.

### Step 3. User views

We conducted eight FGD with 82 participants. The median age of FGD participants was 17 years with 32 men and 50 women; 39 were from London, eight from Manchester and 35 from rural Cambridgeshire (see Additional file 3). Participants wanted the questionnaires to be as short as possible. They had no objections to the prototype questionnaire design and content. They reported that the study materials should be identifiable only to them and suggested using a coloured postal envelope. They thought that the simplified instructions were clear. Participants asked to receive a text or phone call before we sent the materials so they would know to look out for them. They were concerned that sending materials by recorded delivery could call attention to the post and that parents would ask questions. Women approved of the vaginal swab only, rather than providing a choice between swab and urine sample.

### Final follow-up procedures

The results of steps 1–3 informed the final follow-up procedures.

#### Materials

##### Questionnaires

Our follow-up questionnaire was two pages long. Research evidence and feedback from the target group suggests that questionnaires should be as short as possible [7, 9–11, 16]. In accordance with guidance from our consultation with experts and evidence from Edwards et al. (2009), we ordered the key questions on treatment and sexual behaviour first [7]. We did not include personal details on the questionnaires and included a statement about confidentiality [7]. We offered an online questionnaire as an alternative to postal completion. Participants had the opportunity to reply to key questions by text and email if they had not responded after the final paper mailing.

##### Postal test kit

We selected a postal test kit that would fit through the smallest letterbox that we measured. The kits contained only essential components (see Additional file 4). We included a swab only for women. We used the simplified instruction slips. We used a pared-down laboratory slip that only required participants to write the date the sample was collected. Participants had the option of providing their test sample at the clinic.

##### Letters

The letters were as short as possible [7, 9–11, 16] (see Additional file 5 for a sample follow-up letter). The template was formal but the tone was casual [16]. The letters included a statement saying that the recipient was helping to improve the health of young people, a National Health Service/National Institute of Heath Research logo and the Trial Coordinator’s University address [7].

##### Envelopes and postage

We sent all correspondences in blue envelopes and used first class outward and incoming postage [7, 16]. We did not send the post by recorded delivery or add a ‘teaser’ on the envelope (a ‘teaser’ is a statement indicating that that there may be a benefit to opening) because of its potential to call attention to participants’ participation in the study, which could compromise confidentiality [7].

##### Mailings and incentives

We notified all participants by text message before the initial mailing of the questionnaire and test kit [7, 16]. All initial mailings of the questionnaire and test kit included £5 unconditional cash incentive [7, 10, 12]. We sent £20 cash to all participants who returned the chlamydia test sample [7, 8, 12, 16]. While there is no evidence from systematic reviews for sending additional cash after receipt, our experience in the txt2stop trial was that additional participants did respond to this [17]. We contacted non-responders by phone, text messages and email, unless they opted out of further follow-up at any stage [7–9, 16].

#### Month 1 questionnaire

The initial questionnaire posting included a £5 cash unconditional incentive [7, 10, 12]. We sent an email message, which included a link to the online questionnaire, within a week after the initial questionnaire posting [7–9, 16]. We posted the questionnaire again, 2 to 3 weeks later, and sent a second email within a week after this [7, 16]. The third paper mailing included a statement in the letter saying that we would send an additional £10 if we received it within 2 weeks [7, 8, 12, 16]. The fourth paper mailing included a statement in the letter that we would enter participants into a £50 prize draw if they returned the questionnaire within 2 weeks [7, 8, 12, 16]. All responders were eligible for the prize draw. Finally, we emailed, texted and posted key outcome questions to non-responders [7–9, 16].

#### Month 3 chlamydia test

The initial postal test kit included a £5 cash unconditional incentive [7, 10, 12]. All letters mentioned that they would receive £20 if they returned the sample [7, 8, 12, 16]. We sent the test kit to non-responders a further three times [7, 16]. The fourth mailing included a statement in the letter saying that they would be entered into a prize draw for £50 if we received it within 2 weeks [7, 8, 12, 16]. At each mailing, we followed up with participants by phone and email [7–9]. We sent the test kit to non-responders once a month [7, 16].

#### Month 12 questionnaire and chlamydia test

We sent the initial 12-month questionnaire and test kit together with a £10 cash unconditional incentive [7, 10, 12]. All letters mentioned that they would receive £20 if they returned the sample [7, 8, 12, 16]. The initial letter included a statement saying that we would enter participants into a £50 prize draw if they returned both the questionnaire and test [7, 8, 12, 16]. We phoned and sent an email message, which included a link to the online questionnaire, around 3 weeks after the initial mailing [7–9, 16]. We sent the questionnaire and test kit to non-responders a further three times [7–9, 16]. At each mailing, we followed up with participants by phone and email [7–9]. We sent the test kit to non-responders once a month [7, 16]. We emailed, texted and posted one or two key outcome questions to questionnaire non-responders (according to their chlamydia status at enrolment) [7–9, 16].

### Response

Ninety-two percent (183/200) provided questionnaire outcome data at 1 month, 86 % (171/200) provided a chlamydia test sample at 3 months, 82 % (163/200) provided questionnaire outcome data and 80 % (160/200) provided a chlamydia test sample at 12 months (see Additional file 6).

### User views of the final follow-up procedures

We interviewed 17 of the original 20 main pilot trial interview participants within 2 months of sending the 3-month postal STI testing kits (see Additional file 7). Nine follow-up interview participants tested positive at enrolment. None of the participants had any criticisms of the procedures. They thought that the pre-notification served as a reminder to look in the post. Participants mentioned that the blue envelope helped them recognise that it was study material. One said that the letters were polite in that we were not telling them that they had to send it back and another appreciated that they were short and to the point. Most participants thought that the instructions were easy to follow and most did not have any criticisms of the postal test kit. One participant said that initially they were not clear whether they should post the box on its own or inside an envelope. Another suggested including a urine collection pouch. One participant said that she initially had difficulty opening the swab. Another suggested that we include a condom in the kit.

Most participants said that they would have returned the questionnaire and chlamydia sample even if they were not offered an incentive. Some indicated that the motivating factor was their health rather than the money. A few participants mentioned that the unconditional £5 motivated them to return it and another wanted to return it because we ‘treated’ them and said they would have procrastinated without it. Women preferred the swab sample collection method and no participants mentioned that they would rather have had a choice (swab or urine).

## Discussion

### Summary of the main findings

This paper describes how we developed follow-up procedures for the pilot RCT of our sexual health intervention delivered by text message. We used evidence-based methods, tested prototype procedures and consulted with users. We achieved 92 % (183/200) overall follow-up for the self-reported data at 1 month and 82 % (163/200) at 12 months. We achieved 86 % (171/200) follow-up for the chlamydia test at 3 months and 80 % (160/200) at 12 months.

### Comparisons with other studies

This pilot trial’s follow-up response for return of postal chlamydia test samples is high when compared to similar trials, screening initiatives and collection of self-reported sexual health data [4, 6]. The chlamydia screening studies (‘ClaSS’) project [4, 5] used steps described in this paper such as choosing a test kit that would fit through a ‘standard’ letterbox and testing the kit with the target group [18]. However, the researchers evaluated interventions to increase follow-up after the project. Our response may be higher than the ‘ClaSS’ project because our participants agreed to provide follow-up data when they were recruited, we offered unconditional incentives and included only essential test kit components. The ‘Sexunzipped’ trial used evidence-based methods to increase postal follow-up response [6]. Response in this trial may have benefitted from testing all the trial procedures and consulting with the target group [17].

We developed follow-up procedures in a similar way in the smoking cessation txt2stop pilot and main trial [17, 19]. The pilot trial achieved 96 % response for self-reported data at 1 month and 92 % at 6 months [19]. In the main trial, we achieved 95 % (5524/5800) response for self-reported data and 81 % (542/666) response for postal salivary cotinine tests at 6 months [17]. An earlier trial that did not use a similar approach to develop follow-up procedures only achieved 74 % response for self-reported data collected at 6 months [20].

### Limitations

We describe a single case study using three steps to develop follow-up procedures. The steps are not guaranteed to produce useful information. For example, researchers may identify barriers in step 2, but it may not be possible to overcome the barriers when resources are limited. Consulting with the target group could be challenging when administering a multi-site international trial from a central location. We did not test the kits with young people in step 2, which could have been beneficial. Our search for strategies to increase postal follow-up involved searching the Cochrane Library only. Researchers who adopt our method could benefit from searching a range of databases.

### Implications of findings

Our case study suggests that our approach could increase follow-up in trials. Previous work has highlighted the problems with missing data, and its reporting and handling [1, 21]. Our approach aims to boost the number of outcome events recorded, minimising bias and increasing statistical power. An advantage of this approach is that researchers can make fewer post-hoc assumptions about outcomes in participants lost to follow-up. Some of the specific follow-up procedures that we used could be relevant to other trials on sensitive topics with young people (such as using coloured envelopes). By designing acceptable and effective follow-up procedures at the outset of trials, researchers could avoid spending time and resources deploying less effective follow-up procedures.

## Conclusions

The approach described in this paper gives researchers an additional tool to minimise losses to follow-up in trials. Our results show that a high follow-up to self-reported data in questionnaires and postal test kits can be achieved, even in sensitive areas such as sexual health.

## Additional files

Additional file 1:
**Key findings and implications for follow-up design.** Details of follow-up strategies where there is evidence of increasing the odds of response. (DOCX 35 kb) Additional file 2:
**Lab diagnostic company’s postal test kit components.** List of components of the lab diagnostic company’s postal test kit. (DOCX 16 kb) Additional file 3:
**Focus group discussion participant characteristics.** Characteristics of the participants who took part in the focus group discussions. (DOCX 12 kb) Additional file 4:
**Final postal test kit components.** List of the final components of the postal test kit used in the pilot trial. (DOCX 15 kb) Additional file 5:
**Sample follow-up letter.** A sample of a follow-up letter sent to pilot trial participants. (DOCX 53 kb) Additional file 6:
**Follow-up response.** Cumulative response at each mailing and response by mode. (DOCX 16 kb) Additional file 7:
**Follow-up interview participant characteristics.** Characteristics of the participants who took part in the follow-up interview study. (DOCX 13 kb)

## Abbreviations

CTU: Clinical Trials Unit
LSHTM: the London School of Hygiene and Tropical Medicine
FGD: focus group discussion
NIHR HTA: National Institute for Health Research Health Technology Assessment
RCT: randomised controlled trial
