# Erratum to: Simple steps to develop trial follow-up procedures

**DOI:** 10.1186/s13063-016-1614-8

**Published:** 2016-10-06

**Authors:** Ona McCarthy, Rebecca S. French, Ian Roberts, Caroline Free

**Affiliations:** 1 Department of Population Health, London School of Hygiene and Tropical Medicine, Keppel St, London, WC1E 7HT UK; 2 Department of Social and Environmental Health Research, London School of Hygiene and Tropical Medicine, 15-17 Tavistock Place, London, WC1H 9SH UK

## Erratum

Unfortunately, the original version of this article [1] contained an error. There were errors in the reference numbers in Additional file 1. This has now been corrected and Additional file 1 is included here with the correct reference numbers.

## Additional file

Additional file 1: Key findings and implications for follow-up design. Details of follow-up strategies where there is evidence of increasing the odds of response. (DOCX 19 kb)
