# Supplementary material for: Simple steps to develop trial follow-up procedures
Source: Trials. 2016 Jan 15;17:28. doi: 10.1186/s13063-016-1155-1 (PMC4714530; doi:10.1186/s13063-016-1155-1)
Supplement: Additional file 2: — Lab diagnostic company’s postal test kit components. List of components of the lab diagnostic company’s postal test kit. (DOCX 16 kb) [file 13063_2016_1155_MOESM2_ESM.docx]

1. Urine sample tube
2. Urine collection pouch (male and female)
3. Vaginal swab tube (female)
4. Lab request form

(type of sample, date collected, GP information (optional), first and last name, date of birth, sex, postcode. ethnicity, mobile number, confirmation of mobile number, email address and full postal address)

1. Instructions

(Two sides of a 23.5cm x 18cm sheet of paper. One side displayed an ‘A, B, C’ step process of how to provide the sample and post the box, images, information about Chlamydia along with company branding and web addresses. The other side of the sheet was divided into two columns. The left side column was coloured pink, included ‘WOMEN- vaginal swab’ at the top and text and pictorial instructions on how to provide a vaginal swab sample. The right side column was coloured blue and included ‘MEN- urine sample’ at the top and text and pictorial instructions on how to provide a urine sample.)

1. Chlamydia information leaflet
2. Business promotional card
3. Condom
4. Pen
