# Supplementary material for: Simple steps to develop trial follow-up procedures
Source: Trials. 2016 Jan 15;17:28. doi: 10.1186/s13063-016-1155-1 (PMC4714530; doi:10.1186/s13063-016-1155-1)
Supplement: Additional file 3: — Focus group discussion participant characteristics. Characteristics of the participants who took part in the focus group discussions. (DOCX 12 kb) [file 13063_2016_1155_MOESM3_ESM.docx]

| **Characteristic** | **N (%)** |
| --- | --- |
| **Age (years)** |  |
| 16-19 | 45 (55) |
| 20-24 | 15 (18) |
| No data | 22 (27) |
| **Gender** |  |
| Female | 50 (61) |
| Male | 32 (39) |
| **Sexual orientation** |  |
| Heterosexual | 30 (37) |
| Bisexual | 3 (4) |
| Gay/Lesbian | 1 (1) |
| No data | 48 (59) |
| **Ethnicity** |  |
| White British/White other | 45 (55) |
| Black/Black British | 18 (22) |
| Asian British | 1 (1) |
| Mixed | 6 (7) |
| Other | 1 (1) |
| No data | 11 (1) |
| **Education/work** |  |
| School | 2 (2) |
| College/university | 37 (5) |
| Working | 6 (7) |
| Unemployed | 5 (6) |
| Long term sick | 1 (1) |
| No data | 31 (38) |
