# Supplementary material for: Simple steps to develop trial follow-up procedures
Source: Trials. 2016 Jan 15;17:28. doi: 10.1186/s13063-016-1155-1 (PMC4714530; doi:10.1186/s13063-016-1155-1)
Supplement: Additional file 4: — Final postal test kit components. List of the final components of the postal test kit used in the pilot trial. (DOCX 15 kb) [file 13063_2016_1155_MOESM4_ESM.docx]

1. Urine sample tube (male only)
2. Vaginal swab tube (female only)
3. Lab request form (participants only required to write date)
4. Instructions (simplified)
