# Supplementary material for: Simple steps to develop trial follow-up procedures
Source: Trials. 2016 Jan 15;17:28. doi: 10.1186/s13063-016-1155-1 (PMC4714530; doi:10.1186/s13063-016-1155-1)
Supplement: Additional file 5: — Sample follow-up letter. A sample of a follow-up letter sent to pilot trial participants. (DOCX 53 kb) [file 13063_2016_1155_MOESM5_ESM.docx]

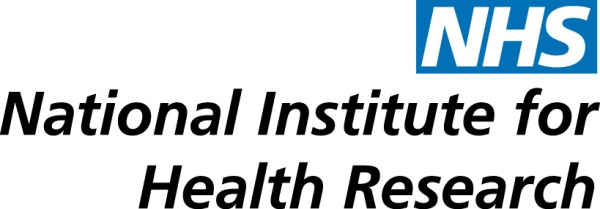


DATE

FIRST & LAST NAME

ADDRESS

Hi FIRST NAME,

Thanks for taking part in the texting study. You’re helping to improve health of young people.

It’s now 12 months since you joined and time to collect your **final questionnaire & chlamydia test sample.**

1. **Chlamydia test**

Follow the instructions in the brown box to provide your sample. Then send the prepaid box to the lab (just send the box itself- you don’t need to put it in an envelope).

1. **Questionnaire**

Fill in the questionnaire and send it back to me in the stamped envelope. Your answers are really important for the study.

**I’ve included £10 as a thank you for doing #1 and #2**

**I’ll send you £20 more when we receive the sample**

**If you return both you’ll be entered into a PRIZE DRAW to win £50**

As always, your results will remain confidential.

Thank you and I hope you’ve enjoyed taking part!

Ona McCarthy

If you have any questions, please get in touch:

020 7927 2581 or 07970693949

[ona.mccarthy@lshtm.ac.uk](mailto:ona.mccarthy@lshtm.ac.uk)

University of London, Keppel St, London, WC1E 7HT
