# Supplementary material for: Simple steps to develop trial follow-up procedures
Source: Trials. 2016 Jan 15;17:28. doi: 10.1186/s13063-016-1155-1 (PMC4714530; doi:10.1186/s13063-016-1155-1)
Supplement: Additional file 6: — Follow-up response. Cumulative response at each mailing and response by mode. (DOCX 16 kb) [file 13063_2016_1155_MOESM6_ESM.docx]

| Follow-up  time point | Overall  response | Clinic completion response | Postal  response | Online  response | Email^**^  Response | Text^**^  response | Cumulative response at each mailing | | |
| --- | --- | --- | --- | --- | --- | --- | --- | --- | --- |
| Month 1 (questionnaire) | 92%**^*^**  (183/200) | 4%  (7/200) | 73%  (146/200) | 12%  (23/200) | 0.5%  (1/200) | 3%  (6/200) | ***2^nd^*** | ***3^rd^*** | ***4^th^*** |
|  |  |  |  |  |  |  | 64%  (127/200) | 79%  (158/200) | 87%  (174/200) |
| Month 3  (postal test) | 86%**^*^**  (171/200) | 2%  (4/200) | 84%  (167/200) | n/a | n/a | n/a | ***2^nd^*** | ***3^rd^*** | ***4^th^*** |
|  |  |  |  |  |  |  | 59%  (118/200) | 72%  (144/200) | 81%  (161/200) |
| Month 12 (questionnaire) | 82%**^*^**  (163/200) | 2%  (3/200) | 77%  (153/200) | 3%  (5/200) | 0%  (0/200) | 1%  (2/200) | ***2^nd^*** | ***3^rd^*** | ***4^th^*** |
|  |  |  |  |  |  |  | 58%  (116/200) | 66%  (132/200) | 71%  (141/200) |
| Month 12  (postal test) | 80%**^*^**  (160/200) | 2%  (3/200) | 79%  (157/200) | n/a | n/a | n/a | ***2^nd^*** | ***3^rd^*** | ***4^th^*** |
|  |  |  |  |  |  |  | 58%  (115/200) | 66%  (131/200) | 72%  (144/200) |

**^*^**Overall response is greater due to response post 4th mailing

**^**^**To key questions
