# Supplementary material for: Simple steps to develop trial follow-up procedures
Source: Trials. 2016 Jan 15;17:28. doi: 10.1186/s13063-016-1155-1 (PMC4714530; doi:10.1186/s13063-016-1155-1)
Supplement: Additional file 7: — Follow-up interview participant characteristics. Characteristics of the participants who took part in the follow-up interview study. (DOCX 13 kb) [file 13063_2016_1155_MOESM7_ESM.docx]

|  | London | Cambridgeshire |  |
| --- | --- | --- | --- |
|  |  |  |  |
| Age group | Male Female | Male Female | Allocation |
|  |  |  |  |
| 16-18 | 2 1 | 0 1 | Intervention: 14  Control: 3 |
|  |  |  |  |
| 19-21 | 2 3 | 1 2 |  |
|  |  |  |  |
| 22-24 | 1 2 | 1 1 |  |
|  |  |  |  |
