# Supplementary material for: Erratum to: Simple steps to develop trial follow-up procedures
Source: Trials. 2016 Oct 6;17:484. doi: 10.1186/s13063-016-1614-8 (PMC5054543; doi:10.1186/s13063-016-1614-8)
Supplement: Additional file 1: — Key findings and implications for follow-up design. Details of follow-up strategies where there is evidence of increasing the odds of response. (DOCX 19 kb) [file 13063_2016_1614_MOESM1_ESM.docx]

| Steps | Key findings | Implications for follow-up design |
| --- | --- | --- |
| 1. Identify effective strategies to increase follow-up*   (7-12, 16) | Providing monetary incentives  (7) OR 1.99, 1.81 to 2.18  (8) RR 1.18, 1.09 to 1.28  (8) RR 1.12, 1.04 to 1.22 (GBP 10 vs 20)  (16) OR 2.02, 1.79 to 2.27 | We included £5 cash with the initial postal request for the questionnaires and samples.  We sent £20 cash to the participant on receipt of sample. |
|  | Sending post recorded delivery  (7) OR 2.04, 1.60 to 2.61  (8) RR 2.08, 1.11 to 3.87  (16) OR 2.21, 1.51 to 3.25 | We did not send study materials by recorded delivery as this may have compromised confidentiality. However, we sent all materials using first class postage and included a first class stamp on the self-addressed return envelope. |
|  | Adding a ‘teaser’ comment on the envelope  (7) OR 3.08, 1.27 to 7.44 | We did not a teaser comment on the envelope as this may have compromised confidentiality. |
|  | Pre-notifying participants to expect the questionnaire or postal test kit  (7) OR 1.50, 1.29 to 1.74  (16) OR 1.54, 1.24 to 1.92 | We notified participants before we sent the initial questionnaires and postal test kits. |
|  | Personalised letters  (16) OR 1.16, 1.06 to 1.28 | We addressed participants by their first name in letters, email and text messages. |
|  | Coloured ink  (16, 7) OR 1.39, 1.16 to 1.67 | Questionnaires were on white paper, had a light blue colour scheme and black ink. |
|  | Following up with participants after initial request  (7) OR 1.44, 1.25 to 1.65  (8) RR 1.43, 1.22 to 1.67  (9) OR 3.71, 2.30 to 5.97 | We contacted questionnaire non-responders and test kit non-responders. |
|  | Using a short questionnaire  (16) OR 1.86, 1.55 to 2.24  (7) OR 1.73, 1.47 to 2.03  (9) OR 1.35, 1.19 to 1.54 | We designed the questionnaire to be as short as possible by only collecting essential data. |
|  | Providing unconditional incentives  (7) OR 1.61, 1.27 to 2.04  (16) OR 1.71, 1.29 to 2.26 | We included £5 cash with the initial postal request for the questionnaires and samples. |
|  | Providing a second questionnaire & test kit  (7) OR 1.51, 1.13 to 2.00  (16) OR 1.41, 1.02 to 1.94 | We posted the questionnaire four times and sent the website questionnaire link twice. We send the test kit four times and continued to send one each month to non-responders. |
|  | Mentioning an obligation to respond  (7) OR 1.61, 1.16 to 2.22 | We considered mentioning an obligation to respond in the letters but decide against it because our sexual health expert group thought that the target group may respond negatively. |
|  | Stamped return envelopes  (7) OR 1.29, 1.18 to 1.42  (16) OR 1.26, 1.13 to 1.41 | We sent self-addressed, stamped return envelopes for participants to return the questionnaire. Postal test kits included a prepaid box. |
|  | Assurance of confidentiality  (7) OR 1.33, 1.24 to 1.42 | We included the study identification number only on the questionnaire and included a statement about confidentiality on both the questionnaire and letter. |
|  | First class outward mailing  (16, 7) OR 1.12, 1.02 to 1.23 | We sent all materials using first class postage. |
|  | Beginning questionnaires with general questions  (7) OR 0.80, 0.67 to 0.96 | We ordered the key questions about treatment and sexual behaviour first. |
|  | Offering the opportunity to opt out  (7) OR 0.76, 0.65 to 0.89 | We did not include a statement about opting out in the letter. |
|  | Mentioning university sponsorship  (7) (5)OR 1.32, 1.13 to 1.54  (16) OR 1.31, 1.11 to 1.54 | We included OM’s University of London address on all letters. |
| 1. Identify barriers to follow-up completion by testing the process | ***Test kits:*** | |
|  | Some test kits do not fit through letter boxes | We selected a test kit that fit through the smallest letter box. |
|  | The test kit included potentially distracting and non-essential components. | We removed the following components from the test kit: the pen, condom, business promotional card, Chlamydia information leaflet, urine sample tube (for women) and personal details form. |
|  | Most people could not open or broke the urine collection pouch that was included in the test kit. | We removed the urine pouch and tested alternatives such as a urine collection cone and collecting urine directly in the sample tube |
|  | The test kit included an instruction slip, which was divided into two columns- one blue for male urine sample and one pink for the female swab and included non-essential graphics and information. | We simplified and shortened the instruction slip. The revised slip was separated into two- one for the urine and one for the swab sample. The slips were plain white and included only the basic steps- three steps for the urine and four for the swab. |
|  | The test kit required the person to apply a lab tracking label. | We applied the lab tracking label before we sent the test kits. |
|  | The test kit required the person to tick whether they were providing a urine or swab sample. | We ticked urine for men and swab for women before we sent the test kits. |
|  | The pouch for urine collection included in the test kit was unnecessary. | We did not include a pouch or any other container for urine collection. |
|  | Providing only a swab, rather than a choice between a swab and urine was acceptable for women. | We included only swab in test kit for women. |
|  | ***Questionnaire and letters:*** | |
|  | It’s unappealing to order the less relevant questions first. | We ordered the key questions about treatment and sexual behaviour first. |
|  | A long questionnaire is unappealing. | We included necessary questions only. |
|  | Including personal identifiable information on the postal questionnaire could cause confidentiality concerns. | We included the study identification number only on the questionnaire and included a statement about confidentiality on both the questionnaire and letter. |
|  | The letters should include statement about importance of participating. | We included a statement about how participants are helping to improve the health of young people. |
|  | The questionnaire should be short. | We included necessary questions only. |
| 1. Consult with the target group | Young people wanted to be contacted before we sent the kits and questionnaire so they knew to expect it. | We contacted participants before we sent them any study materials. |
|  | The envelopes should be identifiable to participants but not to anyone else. | We sent all study materials in blue envelopes. We alerted participants to this when we contacted them before sending. |
|  | Sending materials by recorded delivery could compromise confidentiality. | We sent self-addressed, stamped return envelopes for participants to return the questionnaire. |
|  | Providing only a swab, rather than a choice between a swab and urine was acceptable for women. | We only included a swab in the test kit for women. |
|  | The simplified and shortened instruction slip that we wrote was clear and acceptable. | We included the simplified and shortened instruction slip rather than the pink and blue graphical instruction slip. |

*All confidence intervals in the table are at the 95% level^[[1]](#endnote-1)^

1. [↑](#endnote-ref-1)
